# Supplementary material for: Can use of the serum anti-PLA2R antibody negate the need for a renal biopsy in primary membranous nephropathy?
Source: PLoS One. 2023 Feb 24;18(2):e0281726. doi: 10.1371/journal.pone.0281726 (PMC9955960; doi:10.1371/journal.pone.0281726)
Supplement: Attachment — Submitted filename: Response to Reviewers final.docx [file pone.0281726.s001.docx]

# Points raised by reviewers with author amendments:

1) Please ensure that your manuscript meets PLOS ONE's style requirements, including those for file naming

**Amendments:**

All PLoS One Manuscript and format guidance followed

2) In ethics statement in the manuscript and in the online submission form, please provide additional information about the patient records/samples used in your retrospective study. Specifically, please ensure that you have discussed whether all data/samples were fully anonymized before you accessed them and/or whether the IRB or ethics committee waived the requirement for informed consent. If patients provided informed written consent to have data/samples from their medical records used in research, please include this information.

**Amendments:**

Added the following sentence in the manuscript under ethics section

The ethics committee therefore waived the requirement for informed consent and no consent or regulatory research approvals were required or obtained prior to this project commencing.

3) Thank you for stating the following financial disclosure:

No funding

At this time, please address the following queries:

a) Please clarify the sources of funding (financial or material support) for your study. List the grants or organizations that supported your study, including funding received from your institution. 
b) State what role the funders took in the study. If the funders had no role in your study, please state: “The funders had no role in study design, data collection and analysis, decision to publish, or preparation of the manuscript.”
c) If any authors received a salary from any of your funders, please state which authors and which funders.
d) If you did not receive any funding for this study, please state: “The authors received no specific funding for this work.”

Please include your amended statements within your cover letter; we will change the online submission form on your behalf.

**Amendments:**

Added this sentence under section 6 (The authors received no specific funding for this work) and amended the cover letter

4) Thank you for stating the following in your Competing Interests section:

No Authors have competing interests

Please complete your Competing Interests on the online submission form to state any Competing Interests. If you have no competing interests, please state "The authors have declared that no competing interests exist.", as detailed online in our guide for authors at <http://journals.plos.org/plosone/s/submit-now> 
 This information should be included in your cover letter; we will change the online submission form on your behalf.

**Amendments:**

Amended on the cover letter

5) Please note that in order to use the direct billing option the corresponding author must be affiliated with the chosen institute. Please either amend your manuscript to change the affiliation or corresponding author or email us at [plosone@plos.org](mailto:plosone@plos.org) with a request to remove this option.

**Amendments:**

Affiliations amended

6) Since the study is limited by the variability in the timing of biopsies and of antibody testing and by nuance in individual biopsies of MN that may impact accuracy of serum-based testing, the conclusion of the paper should be less categorical but focusing to the importance of the results in the proceedings of MN diagnosis.

**Conclusion amended**

To

We have shown that a positive serum anti-PLA_2_R antibody test has high specificity for PMN. This has the potential to allow for the diagnosis and treatment in up to 75% of PMN cases without the need for a renal biopsy. Patients with negative PLA_2_R-Ab tests will still require a biopsy to confirm their diagnosis, and further screening for secondary causes of MN should also be considered in specific cases. But in a subset of patients, the ability to avoid an invasive procedure can provide a benefit to the patients journey and allow for significant cost savings to healthcare providers.

First reviewer’s questions:

1) Methods: due to reduced sample size, it should be added if normal distribution was checked for

Added this sentence in the statistical analysis section:

Normality checked using shapiro-wilk test, and as all parameters significantly deviated from a normal distribution, continuous data are presented as median (interquartile range) and categorical data presented as number and percentage.

2) Methods: was sample size calculation performed?

As this was a retrospective single centre analysis, the sample size was determined by the data we had available to us. We have not conducted any hypothesis tests, but we have added in 95% confidence interval estimates to clearly convey the uncertainty around our estimates.

The manuscript has been updated to reflect this. The receiver operating characteristic (ROC) curve was generated using established methods and the diagnostic sensitivity and specificity of the anti-PLA2R antibody test were calculated, using renal biopsy as the diagnostic reference standard (9,10,11,12,13).  95% confidence intervals (CI) calculated using Wilson procedure without correction for continuity

Ref – Website [http://vassarstats.net/prop1.html](https://urldefense.com/v3/__https:/eur03.safelinks.protection.outlook.com/?url=http*3A*2F*2Fvassarstats.net*2Fprop1.html&data=04*7C01*7CA.F.C.Smith*40leeds.ac.uk*7Cda46e991cca54dd0178b08da11911819*7Cbdeaeda8c81d45ce863e5232a535b7cb*7C1*7C0*7C637841612521931631*7CUnknown*7CTWFpbGZsb3d8eyJWIjoiMC4wLjAwMDAiLCJQIjoiV2luMzIiLCJBTiI6Ik1haWwiLCJXVCI6Mn0*3D*7C1000&sdata=NCTyMpEeWS3SuijvF4odpaC2PCGRc*2BRN8ZYYpHfNf0c*3D&reserved=0__;JSUlJSUlJSUlJSUlJSUlJSUl!!OL9ShTEEVu4!5HR4EwCBPZjmW_uNSZk59Tf5ZBzJRoDBfp7V6UWFlRrSXs1bBuBeiSks3brHjB_L3Gg$) accessed on 29/03/22

And the references from the website added:

Newcombe, Robert G. "Two-Sided Confidence Intervals for the Single Proportion: Comparison of Seven Methods," *Statistics in Medicine,* 17, 857-872 (1998).
Wilson, E. B. "Probable Inference, the Law of Succession, and Statistical Inference," *Journal of the American Statistical Association,* 22, 209-212 (1927).

3) Methods: was diagnosis at biopsy confirmed among different operators?

All biopsies were reported by a Renal Histopathologist and discussed at the departmental biopsy review MDT.

4) Methods: do authors think thanks these results may be of clinical utility?

We believe that those results will be a steppingstone to move away from performing renal biopsies for patients with a positive PLA2R ab test

5) Discussion: do authors think they enrolled enough patients?

As far as we are aware, this is the largest retrospective study to address that question. Given that MN is listed on the National Registry of Rare Kidney Disease (RaDaR), so we think that the number of patients enrolled to answer our question was enough

Second reviewer’s questions:

1)Please elaborate on how primary MN (PMN) is defined?

PMN was confirmed based on a renal biopsy, having a positive anti PLA_2_R ab test and negative secondary work up.

2)Patient's records were reviewed by nephrologists but did each patient have complete workup to exclude potential causes of secondary MN?

Secondary workup for cancers was only performed for certain high-risk categories (older than 50 years old, smokers, history of change in bowel habits and signs of iron deficiency anaemia), atypical findings on histology or based on full history and examination. PSA for those >50 years old and CT TAP/colonoscopy and virology screen were done only when clinically indicated. Autoimmune screen was only performed if other autoimmune conditions were present. The most recent evidence from KDIGO 2021 is still to perform a secondary work up regardless the antibody test result.

3)Were histopathologic features of kidney biopsy considered (e.g. immunofluorescence staining pattern, location(s) of immune deposits, presence of other concomitant renal lesions)?

Our findings were not specifically correlated with histological features on biopsy other than the diagnosis of MN itself and we did not stain for PLA2R1 antigen on the renal tissue

4) Any data on concordance rate of serum PLA2R-ab vs. tissue PLA2R staining on biopsy specimen? Any cases exhibiting "immune sink" phenomenon?

We did not stain for PLA2R1 antigen on the renal tissue to correlate that with serum antibody test.

5) Renal biopsies are obtained for both diagnostic and prognostic purposes. Thus, it would be worthwhile to investigate the performance of serum PLA2R-Ab while considering other parameters (e.g. %global and segmental glomerulosclerosis, % tubulointerstitial scarring, tubulointerstitial inflammation, Ehrenreich-Churg stage of MN)

This would be beyond the scope of our study. We agree that this might be of useful prognostic value, but not for diagnostic purposes. Our study was mainly focusing on the diagnostic accuracy of the antibody test.
